# Monthly Energy, Exergy, Environmental, and Economic Performance and Green Hydrogen Production Analysis of a Flat‐Plate Solar Collector‐Driven Organic Rankine Cycle System Under Variable Mass Flow and Irradiance

**DOI:** 10.1002/open.70238

**Published:** 2026-07-01

**Authors:** Ayhan Atiz, Ismail Bozkurt, Mehmet Karakilcik

**Affiliations:** ^1^ Department of Physics Faculty of Science and Letters Cukurova University Adana Turkey; ^2^ Department of Mechanical Engineering Faculty of Engineering Adıyaman University Adıyaman Turkey

**Keywords:** electricity and hydrogen generation, energetic and exergetic efficiency, organic Rankine cycle (ORC), solar collectors, solar radiance, thermal energy

## Abstract

This article comprehensively evaluates the energy, exergy, environmental, and economic performance of a hybrid system integrating a proton exchange membrane (PEM) electrolyzer with an organic Rankine cycle (ORC) driven by flat‐plate solar collectors (FPSCs) (224.64 m^2^) under varying flow rates. Five flow rates were simulated in engineering equation solver (EES), yielding daily electricity outputs between 86.33 MJ and 91.52 MJ, equivalent to 2.676–2.837 GJ throughout July. Hydrogen production ranged from 414.35 to 439.30 g per day, resulting in 12.85–13.62 kg over the month. The highest hourly energy efficiencies varied from 23.82% to 21.71%, while the maximum exergetic efficiencies remained nearly constant at 6.70%–6.72%, indicating stable system behavior. The hybrid setup reduced CO_2_ emissions by 39.39–42.56 kg per day, totaling 1221.09–1984.31 kg in July. While the financial gain declined marginally as the flow rate increased from 10.90 USD to 10.15 USD per day, corresponding to 337.91–314.57 USD monthly, the system nevertheless indicated considerable operational and ecological advantages. The average monthly electricity generation across the evaluated flow rates was calculated as 1.974 GJ, corresponding to an average monthly revenue of 235.12 USD and an estimated annual revenue of 2,821 USD, with a simple payback period of 9.73 years.

## Graphical abstract

The feasibility of using a hybrid flat‐plate solar collector–organic Rankine cycle–proton exchange membrane system for the generation of electricity, heat, and hydrogen simultaneously is analyzed. The energy efficiency of the solar thermal system reaches 23.82%, and hydrogen production of up to 13.62 kg/month is achieved while reducing CO_2_ emissions considerably. The proposed hybrid multigeneration concept proves to be efficient, sustainable, and environmentally friendly for zero‐carbon energy generation.

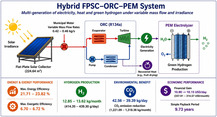

**Abbreviations**
EES: Engineering Equation Solver
ETC: Evacuated tube collector
FPSC: Flat Plate Solar Collector
GWP: global warming potential
HHV: higher heating value
LCOE: levelized cost of electricity
ORC: Organic Rankine cycle
PEM: Proton Exchange Membrane
PV‐T: Photovoltaic‐Thermal
R134a: Refrigerant 134a
RBP: Return payback period

## 1 Introduction

Rapid industrialization and population expansion are driving up global energy consumption, which demands the development of environmentally friendly and low‐carbon energy alternatives. Petroleum and coal continue to account for a large percentage of this demand, resulting in major ecological problems such as carbon emissions, global warming, climate instability, drought, and natural disasters. This necessitates the development of ecofriendly, sustainable, and renewable energy plants. In this context, solar energy stands out among renewable energy resources due to its endless and broad availability. The transition to renewable and sustainable energy resources is a strategic step that benefits not only the environment but also financial stability, energy security, and a habitable environment for the generations to come [1]. Solar energy can be effectively used to produce both electricity and thermal energy. Photovoltaic cells convert solar radiation directly into electricity, while thermal collectors transform it into heat for various applications. Furthermore, producing hydrogen from solar energy offers an environmentally friendly alternative to fossil fuels for energy storage and transportation, positioning solar energy as both an immediate and future energy source.

A flat‐plate solar collector (FPSC) is a traditional collector that transforms solar energy into thermal energy. While primarily intended for residential hot water, the heat generated by FPSCs can also be used for power production in low‐temperature systems [2]. An organic Rankine cycle (ORC) is one such technology that converts low‐temperature thermal energy into electricity using a low‐boiling‐point working fluid [3]. Refrigerant 134a (R134a) is a commonly used working fluid in ORCs due to its favorable properties [4]. Studies have shown that the type of working fluid and its thermal characteristics significantly affect ORC efficiency, with R134a demonstrating high energy output capability at low operating temperatures [5]. Increasing turbine inlet pressure can improve ORC thermal efficiency by up to 14% when using R134a [6]. However, efficiency declines beyond a certain pressure, and R134a performs well in low‐temperature systems [7]. A 1 kW small‐scale ORC achieved 3.33% thermal efficiency at 1.25 MPa and 67.7°C [8]. Two‐stage ORC configurations perform better than single‐stage cycles at higher temperatures [9]. A solar‐powered small‐scale ORC achieved 4.33% energy efficiency at 67.9°C and 1.38 MPa [10]. Increasing the inlet temperature from 55°C to 85°C and evaporator pressure from 400 to 600 kPa yielded a maximum thermal efficiency of 3.907% [11]. Raising the source temperature from 80°C to 110°C increased R134a thermal performance from approximately 8.7% to 11.5% [12]. At 130°C evaporator temperature and 10 bar pressure, thermal performance was calculated as 3.43% [13]. An ORC using a CO_2_/R134a mixture achieved a maximum energy efficiency of 10.14% [14]. Using low‐temperature brine (27°C) as a heat source, the system generated 0.917 kW of electrical power with a thermal efficiency of 1.46% [15]. Increasing turbine inlet pressure from 1 to 4 MPa, R134a outperformed other tested fluids, reaching 27.2% thermal efficiency at 4 MPa [16].

When ORC is integrated with solar‐based technologies, waste heat can be recovered to improve overall efficiency. A photovoltaic‐thermal‐evacuated tube collectors‐ORC (PVT‐ETC‐ORC) hybrid system using R134a achieved an energetic efficiency of 19.76%, with surplus heat used for pool heating and domestic hot water [17]. Another hybrid configuration using FPSCs, an *n*‐butane ORC, and a proton exchange membrane (PEM) electrolyzer produced electricity and hydrogen year‐round, with ORC efficiency up to 5.46% [18]. An experimental study on R134a flow boiling in horizontal micro‐finned tubes reported higher heat transfer coefficients, lower wall temperature differentials, and delayed dry‐out compared to smooth tubes. Increased heat flux improved heat transfer and bubble nucleation, while the helical flow induced by the fin design significantly enhanced convective performance and overall system efficiency [19]. A hermetic scroll expander tested in an R134a ORC at 75–85°C achieved a net thermal efficiency of 6.18% and a maximum power output of 1.33 kW, with excessive superheating reducing performance [20]. A numerical study on supercritical R134a in U‐bent tubes showed that curvature‐induced vortices enhanced heat transfer by up to 42% on average and 29%–49% locally, depending on curvature position and flow conditions [21].

Hydrogen production in solar‐assisted ORCs is crucial for building a sustainable, carbon‐free energy infrastructure, as it addresses solar intermittency by storing energy as hydrogen. An ORC‐based solar hydrogen generation system using an ETC with acetone achieved a hydrogen production rate of 0.000004433 kg/s, with energy and exergy efficiencies of 3.45% and 2.15%, respectively [22]. Another study proposed a solar‐assisted waste‐to‐energy system combining a Brayton cycle, an ORC, and a PEM electrolyzer. The system produced hydrogen at 0.00031 kg/s, with energy and exergy efficiencies of 32.97% and 30.42%, respectively, and an electricity cost of 0.02297 $/kWh [23]. Recent studies have further advanced ORC‐based poly‐generation systems. A poly‐generation system utilizing low‐temperature flue gas waste heat in power plants coupled with an ORC, achieving a total gain output increase of approximately 47% compared to an independent system, with R245fa and R113 yielding the highest performance indicators [24]. A numerical study investigated three novel solar‐driven poly‐generation systems integrating ORC with humidification–dehumidification desalination and desiccant cooling for residential applications, reporting total gain output ratio improvements of 68.5%–95.5% over separated systems, with *n*‐octane and R113 identified as optimal working fluids [25]. Additionally, recent work on water‐based PVT collectors demonstrated that a zigzag semicircular thermal absorber design improved electrical efficiency by 11.97% compared to noncooled photovoltaic (PV) systems while achieving 76.75% thermal efficiency [26].

A system's ability to reduce daily carbon emissions is directly linked to its potential to mitigate global warming. Renewable energy sources promote long‐term environmental preservation by preventing significant carbon emissions [27]. Furthermore, economic viability is essential for global deployment and adoption of such systems [28].

The literature shows that ORC performance is influenced by heat source temperature, evaporator pressure, flow rate, working fluid type, heat exchanger geometry, and system integration. R134a offers excellent low‐temperature evaporation capability, enabling high energy and exergy efficiency even in small‐scale systems. Novel heat exchanger designs, such as micro‐finned tubes and curved vapor generators, can improve heat transfer by up to 40%. Solar‐assisted and waste heat recovery ORC systems have demonstrated simultaneous electricity and hydrogen production, while PEM‐based hybrid configurations generate carbon‐free fuels. A multistage supercritical CO_2_ Brayton‐ORC system achieved energy and exergy efficiencies of 32.97% and 30.42%, with a levelized electricity cost of only $0.02297/kWh. Overall, ORC‐based systems are efficient, sustainable, and economically viable for renewable energy conversion, and their integration with hydrogen production positions them as a key component of future clean energy infrastructure.

Previous studies have not comprehensively investigated the transient daily performance of R134a‐based ORC systems integrated with FPSCs under simultaneously varying mass flow rates, solar irradiance, and ambient temperature conditions. Most earlier studies focused primarily on steady‐state or constant‐flow operation. In contrast, the present study systematically evaluates the hourly performance (09:00–15:00) of the system under five different water mass flow rates (0.42–0.46 kg/s).

The novelty of this study can be summarized in three main contributions:

• For the first time, a combined FPSC–ORC–PEM system is analyzed through detailed hourly thermodynamic, environmental, and economic assessments under variable mass flow conditions and real solar irradiance data for July in Adana.
• Unlike previous studies where hydrogen production is generally considered a secondary output, the PEM electrolyzer in this article is directly integrated with the time‐dependent electrical output of the ORC system, and the corresponding daily and monthly hydrogen production rates (12.85–13.62 kg) are quantified for different mass flow rates.
• The proposed configuration simultaneously integrates low‐temperature fruit drying, residential hot‐water storage, electricity generation, and green hydrogen production within a single solar‐driven system. To the best of the authors’ knowledge, such a multioutput R134a‐based ORC configuration operating under variable flow conditions has not previously been investigated.

Accordingly, the main objective of this study is to evaluate the thermodynamic, economic, and environmental performance of the proposed FPSC–ORC–PEM system under varying operating conditions. Energy and exergy efficiencies, electricity and hydrogen production capacities, economic feasibility, carbon‐emission reduction potential, annual economic contribution, and system payback period were analyzed on hourly, monthly, and annual bases. The annual economic contribution and payback period were estimated using the representative operating data obtained for January and July. In addition, waste heat recovered from the ORC was utilized for low‐temperature fruit drying and domestic hot‐water storage. The results demonstrate the system's potential to support sustainable multigeneration energy applications, reduce fossil fuel dependency, and contribute to the transition toward low‐carbon energy systems.

## 2 System Description

**FIGURE 1:**
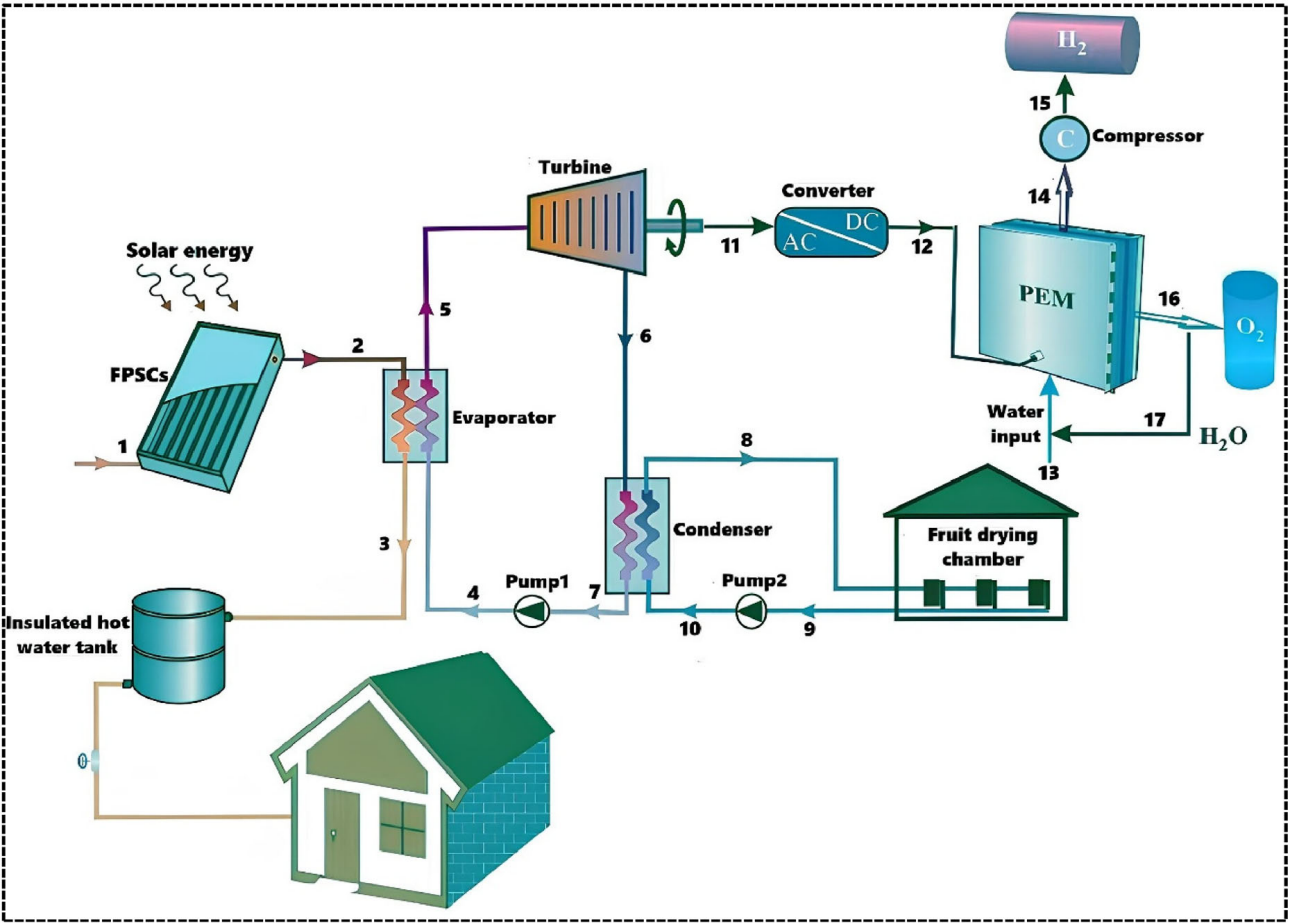
Combined low‐carbon system powered by solar energy.

Figure 1 shows the whole framework, which includes 224.64 m^2^ of FPSCs, an R134a‐powered ORC, a PEM, a fruit‐drying chamber, and a hot water cylinder for residential usage. Water from the municipal network reaches the framework via a changeable valve at point 1 and flows to the flat‐plate collectors, where it gets hot by solar irradiation. After exiting point 2, the heated water goes via the ORC's evaporator before exiting point 3 and into the hot‐water storage tank. The stored water can be used for residential uses as required. The low‐temperature R134a fluid is fed into the evaporator at point 4 of the ORC loop, where it absorbs heat and vaporizes. The resulting vapor departs at point 5 and powers the turbine generator to produce electricity. Mechanical labor is converted into energy when the fluid expands in the turbine, and it exits at point 6. The generated electricity is routed from point 11 to a converter, then to point 12 to the PEM electrolyzer, which produces hydrogen. After R134a leaves the turbine and loses a significant portion of its energy, it reaches the condenser at point 6 and transfers the remaining heat to the water flow entering from point 10. The fruit is dried using the hot water that leaves at point 8. The thermodynamic cycle is completed when the condensed R134a reaches the pump at point 7. Additionally, R134a's thermophysical characteristics (such as its molecular weight, boiling point, and critical temperature) are displayed in Table 1, and the FPSCs’ geometric and thermal characteristics (collector area, absorber plate material, absorptivity, emissivity, etc.) are shown in Table 2. These parameters outline the crucial input information that is utilized in the energy and exergy assessments.

**TABLE 1:** Thermophysical properties of R134a [29].

Property	Value
Molecular mass (g/mol)	102.03
Boiling point (°C)	−26.1
Critical temperature (°C)	101
Critical pressure (MPa)	4.056
ASHRAE 34 safety group	A_1_
Atmospheric life (years)	14
Ozone depletion potential	0
Global warming potential (100 years)	1430

**TABLE 2:** Technical properties of the FPSCs.

Property	Value	Unit
Number of panels	120	—
Length	195	cm
Width	96	cm
Diameter of the tube	6.35	mm
Thickness of plate	0.16	mm
Thickness of glass	1.6	mm
Thickness of thermal insulation matter	40	mm
Tube matter (Copper)	—	—
Absorption coefficient of the absorber surface	0.95	—
Emissivity of the absorber surface	0.13	—

## 3 Assessment Energy and Exergy Efficiency of the System

During the system's construction, a number of particular assumptions were established in order to produce thermodynamic findings that were precise, reliable, and realistic. These presumptions made the study both computationally possible and practically significant by simplifying the system's intricate physical behavior. To make the model theoretically solvable while retaining technical relevance, a few idealized criteria were essentially added. Furthermore, as they improve energy efficiency, maximize resource utilization, and reduce environmental effects, such presumptions are crucial to the design of sustainable energy systems. A summary of the key assumptions is provided below.

a) The ambient temperature and the temperature of the water entering the system are the same.
b) The ambient pressure measures 101.325 kPa.
c) Heat losses in pipes are insignificant.
d) The FPSCs maintain a tilt angle of 10° throughout the day.
e) The hot water storage tank is believed to be of adequate size.
f) Hydrogen enters the storage tank with 98% efficiency.
g) Steady‐state conditions are assumed for all system components during the hourly analysis.

The manuscript relies on several simplifying assumptions, including negligible heat losses, constant component efficiencies, and steady‐state operation. These assumptions inevitably introduce limitations regarding the real‐world applicability of the results. In particular, neglecting pipe heat losses may lead to an overestimation of the thermal energy delivered to the ORC evaporator, especially under large‐scale operation or low ambient temperature conditions. The steady‐state assumption disregards transient effects such as rapid fluctuations in solar irradiance, cloud cover, and the thermal inertia of the collectors, which may cause deviations between predicted and actual system performance. Moreover, assuming constant efficiencies for components such as the turbine and pump does not fully represent off‐design or part‐load operation conditions. Although these simplifications are necessary to ensure model tractability, the obtained results should be interpreted as idealized performance indicators. Future studies incorporating dynamic modeling and experimental validation would improve both the accuracy and the practical relevance of the system evaluation.

### 3.1 Energetic and Exergetic Assessment of the FPSCs

FPSCs transfer solar energy to the working fluid, raising its temperature. The warmer fluid flows into the storage unit and is replaced by cooler water. Under adequate solar radiation, the fluid within the collector absorbs solar energy and keeps its temperature over a particular threshold. The following equations were used to perform energy analysis and determine the collector performance [30].

(1) $${\dot{E}}_{\text{FPSC}}={\dot{E}}_{U}+{\dot{E}}_{\text{loss}}$$

This equation represents the energy balance on the FPSC, where the total incident solar energy is partitioned into useful gains and thermal losses to the environment.

The total solar energy reaching the collector surface is calculated as follows

(2) $${\dot{E}}_{\text{FPSC}}=\dot{E}A_{\text{FPSC}}$$

where $\dot{E}$ represents total solar energy per unit area according to the collector's tilt angle. Accordingly, the energy efficiency of the FPSC (${\text{η}}_{\text{FPSC}}$) is calculated as follows

(3) $${\text{η}}_{\text{FPSC}}=\frac{{\dot{E}}_{U}}{{\dot{E}}_{\text{FPSC}}}$$

In addition, the energetic performance of the FPSC can be referred to in simplified terms as follows [31]:

(4) $${\text{η}}_{\text{FPSC}}=0.70-3.4\frac{\left(T_{m}-T_{0}\right)}{\dot{E}}$$

Accordingly, the exergy performance of the collector is calculated as

(5) $$\dot{E}x_{\text{FPSCs}}=\dot{E}x_{U,\text{FPSCs}}+\dot{E}x_{\text{loss},\text{FPSCs}}+\dot{E}x_{\text{dest},\text{FPSCs}}$$

This exergy balance equation accounts for the useful exergy output, exergy losses to the surroundings, and exergy destruction due to irreversibilities within the collector. In addition, the exergy efficiency of the FPSCs can be calculated as follows

(6) $${\text{ψ}}_{\text{FPSCs}}=\frac{\dot{E}x_{U,\text{FPSCs}}}{\dot{E}x_{\text{FPSCs}}}$$

### 3.2 Energy and Exergy Analyses of the ORC

The working fluid at low, medium, or high temperature from external sources is pumped into the evaporator to enable its vaporization within the ORC system. The ORC turbine generates mechanical power from the vaporized liquid and causes the turbine to rotate, thus producing electrical energy through the generator connected to the turbine [32]. The net electrical energy output (${\dot{W}}_{\mathrm{net},\mathrm{ORC}}$) is expressed as follows [33]:

(7) $${\dot{W}}_{\mathrm{net},\mathrm{ORC}}={\dot{W}}_{G}-\left({\dot{W}}_{\text{pump}1}\right)$$

The net power output of the ORC is obtained by subtracting the power consumed by the pump from the power generated by the turbine‐generator unit. Here, ${\dot{W}}_{\text{pump}1}$ and ${\dot{W}}_{G}$ are given below:

(8) $${\dot{W}}_{\text{pump}1}={\dot{m}}_{4}\left(h_{4}-h_{7}\right)$$

(9) $${\dot{W}}_{G}={\text{η}}_{\mathrm{tur}}{\text{η}}_{G}{\dot{m}}_{5}\left(h_{5}-h_{6}\right)$$

where the product ${\text{η}}_{\mathrm{tur}}$ and ${\text{η}}_{G}$ are accepted as 0.95 for this article. Finally, the following formula can be utilized to compute the ORC cycle's energetic performance:

(10) $${\text{η}}_{\mathrm{ORC}}=\frac{{\dot{W}}_{\mathrm{net},\mathrm{ORC}}}{{\dot{Q}}_{\mathrm{Eva}}}$$

This expression defines the first‐law efficiency of the ORC as the ratio of net electrical power output to the thermal energy input received from the FPSCs via the evaporator. In this context, ${\dot{Q}}_{\mathrm{Eva}}$ denotes the total heat energy transferred to the evaporator from the external source. The following formula is used to obtain this parameter's value:

(11) $${\dot{Q}}_{\mathrm{Eva}}={\dot{m}}_{5}\left(h_{5}-h_{4}\right)$$

Another important parameter is the overall exergetic performance of the ORC, which is determined as [34]:

(12) $${\text{ψ}}_{\mathrm{ORC}}=\frac{{\dot{W}}_{\mathrm{net},\mathrm{ORC}}}{\dot{E}x_{\mathrm{in},\mathrm{ORC}}}$$

The exergy efficiency of the ORC measures how effectively the system converts the available exergy from the heat source into useful work. $\dot{E}x_{\mathrm{in},\mathrm{ORC}}$ denotes the exergy obtained from the internal source for the ORC, which is calculated as:

(13) $$\dot{E}x_{\mathrm{in},\mathrm{ORC}}={\dot{m}}_{2}\left[\left(h_{2}-h_{0}\right)-T_{0}\left(s_{2}-s_{0}\right)\right]$$

### 3.3 Hydrogen Production

PEM electrolyzer is an electrochemical transformation technology that reacts swiftly to dynamic variations in load while operating at low temperatures. This technology uses clean energy to divide water into high‐purity hydrogen and oxygen. The system's small dimensions, excellent productivity, and zero emissions provide it with a potential hydrogen generation technique. Reference [35] provides a formula for calculating a PEM electrolyzer's total energy efficiency (${\text{η}}_{\text{elec}}$).

(14) $${\text{η}}_{\text{elec}}={\text{η}}_{V}{\text{η}}_{F}$$

The electrolyzer has an efficiency of 60%–80%. According to reference [36], electrolyzing one mole of water requires 285.84 kJ of electrical energy, resulting in 2 g of hydrogen and 16 g of oxygen. The PEM electrolysis performance in this study was determined to be 70%.

### 3.4 Energy and Exergy Performance of the System

Crucial performance information is obtained from the system's overall energetic efficiency, which is determined using the first equation of thermodynamics. Furthermore, the second‐law (exergetic) efficiency provides more information about the irreversibilities and actual performance of the system. The overall energetic efficiency is defined as:

(15) $${\text{η}}_{\text{overall},\mathrm{sys}}=\frac{{\dot{Q}}_{\text{heat}}+{\dot{Q}}_{\mathrm{st}}+{\dot{m}}_{H_{2}}h_{H_{2}}+{\dot{m}}_{H_{2}}\text{*HHV}-{\dot{W}}_{\text{comp}}-{\dot{W}}_{\text{pump}2}}{{\dot{E}}_{\mathrm{in}}}$$

This overall energy efficiency equation accounts for all useful outputs (fruit‐drying heat, stored hot water, and hydrogen energy, including its higher heating value) minus parasitic loads (compressor and pump), normalized by the total solar energy input. The expressions for ${\dot{Q}}_{\mathrm{st}}$ and ${\dot{Q}}_{\text{heat}}$ can be written:

(16) $${\dot{Q}}_{\mathrm{st}}={\dot{m}}_{3}\left(h_{3}-h_{0}\right)$$

(17) $${\dot{Q}}_{\text{heat}}={\dot{m}}_{8}\left(h_{8}-h_{9}\right)$$

Moreover, the exergy efficiency of the total system is determined as given formula:

(18) $${\text{ψ}}_{\text{overall}}=\frac{\dot{E}x_{\text{heat}}+\dot{E}x_{\mathrm{st}}+{\dot{m}}_{H_{2}}ex_{H_{2}}+{\dot{m}}_{H_{2}}\text{* HHV}-{\dot{W}}_{\text{comp}}-{\dot{W}}_{\text{pump}2}}{\dot{E}x_{\mathrm{in}}}$$

This overall exergy efficiency equation quantifies the ratio of total useful exergy outputs (fruit‐drying exergy, stored hot water exergy, and hydrogen exergy, including its heating value) minus parasitic loads (compressor and pump) to the total solar exergy input to the system. The expressions for $\dot{E}x_{\text{heat}}$ and $\dot{E}x_{\mathrm{st}}$ can be determined:

(19) $$\dot{E}x_{\text{heat}}={\dot{m}}_{8}\left(ex_{8}-ex_{9}\right)$$

(20) $$\dot{E}x_{\mathrm{st}}={\dot{m}}_{3}\left(ex_{3}-ex_{0}\right)$$

Table 3 lists the thermodynamic properties of the system flow diagram. The table shows the temperature, pressure, and flow rate of the working fluids in both the ORC (R134a) and FPSC (water) circuits at each flow point. These data constitute the fundamental inputs used to determine the system performance. Some temperature values (for instance, *T*_0_ and *T*_2_) are variable, and their precise values are discussed in detail in the discussion and conclusion sections of the article.

**TABLE 3:** Selected thermodynamic variables for the system.

Num.	Parameter	**Temp.,** °C	Pressure, kPa	**Mass flow,** **kg/s**
0	H_2_O	*T*_0_	30	0.42–0.46
00	R134a	*T*_0_	30	0.27–0.30
1	H_2_O	*T*_0_	30	0.42–0.46
2	H_2_O	*T*_2_	150	0.42–0.46
3	H_2_O	*T*_3_	90	0.42–0.46
4	R134a	*T*_7_ + 0.1	1925	0.27–0.30
5	R134a	*T*_2_–5	1925	0.27–0.30
6	R134a	*T*_8_ + 3	975	0.27–0.30
7	R134a	*T*_10_ + 3	975	0.27–0.30
8	H_2_O	*T*_8_	50	0.42–0.46
9	H_2_O	*T*_9_	30	0.42–0.46
10	H_2_O	*T*_9_ + 0.1	30	0.42–0.46

### 3.5 Economic Analyses

The economic assessment of the system reveals its practical value and financial feasibility under real‐world conditions. By evaluating how quickly the system can recover its initial investment, this analysis demonstrates the actual economic benefits of the proposed technology. Accordingly, the payback period (RBP) of the system, excluding labor costs, is calculated as follows [37]:

(21) $$\mathrm{RBP}=\frac{Y}{X-Z-CS_{\mathrm{MD}}}$$

The parameters are found as follows:

(22) $$X=\text{β}{\dot{E}}_{\mathrm{el}}El_{p}$$

(23) $$CS_{\mathrm{MD}}=CS_{m}+CS_{d}$$

where $CS_{m}$ and $CS_{d}$ are obtained as:

(24) $$CS_{m}=0.1{\;*\;CS}_{\mathrm{MD}}$$

(25) $$CS_{d}=\sum Y\times \left[i\times {\left(1+i\right)}^{n}\right]/\left[{\left(1+i\right)}^{n}-1\right]$$

The inflation‐adjusted real interest rate i is obtained as follows [38]:

(26) $$i=\frac{i^{ı}-f}{1+f}$$

### 3.6 The Uncertainty Analysis

The results presented in this study are influenced by uncertainties associated with measurement instruments and input parameters. The following input uncertainties were considered in the analysis: solar irradiance with an uncertainty of ±2%, mass flow rate (*ṁ*) with ±0.5%, ORC overall turbine efficiency with ±2%, pump efficiency with ±2%, and fluid and ambient temperature measurements with ±0.3%.

The overall uncertainties of the energy and exergy efficiencies were determined using a first‐order error propagation approach [39].

(27) $${u_{R}}^{2}=\sum_{i=1}^{n}{\left(\frac{dR}{dx_{i}}*u_{x_{i}}\right)}^{2}$$

## 4 Results and Discussion

**FIGURE 2:**
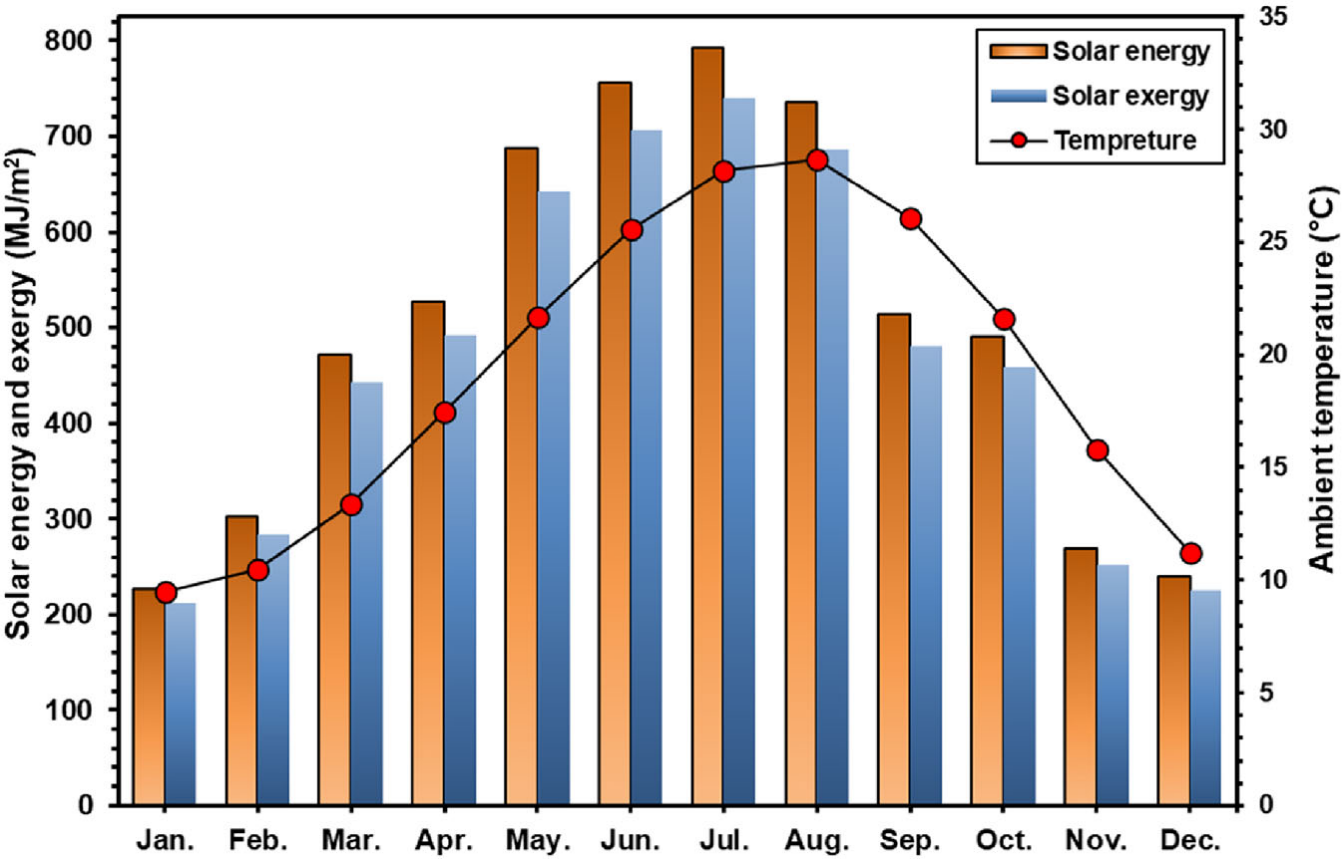
Monthly variation of solar energy and ambient temperature.

In this study, energy and exergy analyses were performed to evaluate the electricity, hydrogen, and hot‐water production performance of a combined system consisting of flat‐plate solar collectors, an ORC, and a PEM electrolyzer for a representative day in July. The combined system is designed to produce hydrogen, thermal energy, and electrical energy. The ORC transformed some of the generated heat energy into electrical power, which was then sent to the PEM to produce hydrogen, while the remaining waste heat was utilized in a fruit‐drying unit and a hot‐water storage tank. This strategy sought to increase overall performance. In addition to thermodynamic efficiency, the investigation examined the combined system's financial results and opportunities for reducing carbon emissions, providing a more thorough assessment of sustainability. Solar energy is the system's principal energy source; therefore, accurately determining the solar radiation reaching the thermal collectors’ surfaces is critical. The Adana Meteorological Station supplied the monthly average ambient temperature and solar radiation data utilized in the present research. The monthly variation of Adana's average ambient temperature and annual average solar energy incident on a horizontal surface is shown in Figure 2. The information, which was gathered from the Adana Meteorological Station, shows how the region's environmental factors and solar potential vary annually. These values served as reference points for the system's energy and performance calculations. July had the highest energy and exergy values (792.66 and 739.53 MJ/m^2^, respectively), while January had the lowest (226.90 and 212.64 MJ/m^2^). August had the highest recorded ambient temperature of 28.7°C, while January had the lowest at 9.5°C. Summer is the best season to use solar power to convert thermal energy into electrical power, as both solar radiation and the surrounding temperature are at their maximum during this time. July's great potential for solar radiation made it the ideal month for thermal‐to‐electric energy conversion.

**FIGURE 3:**
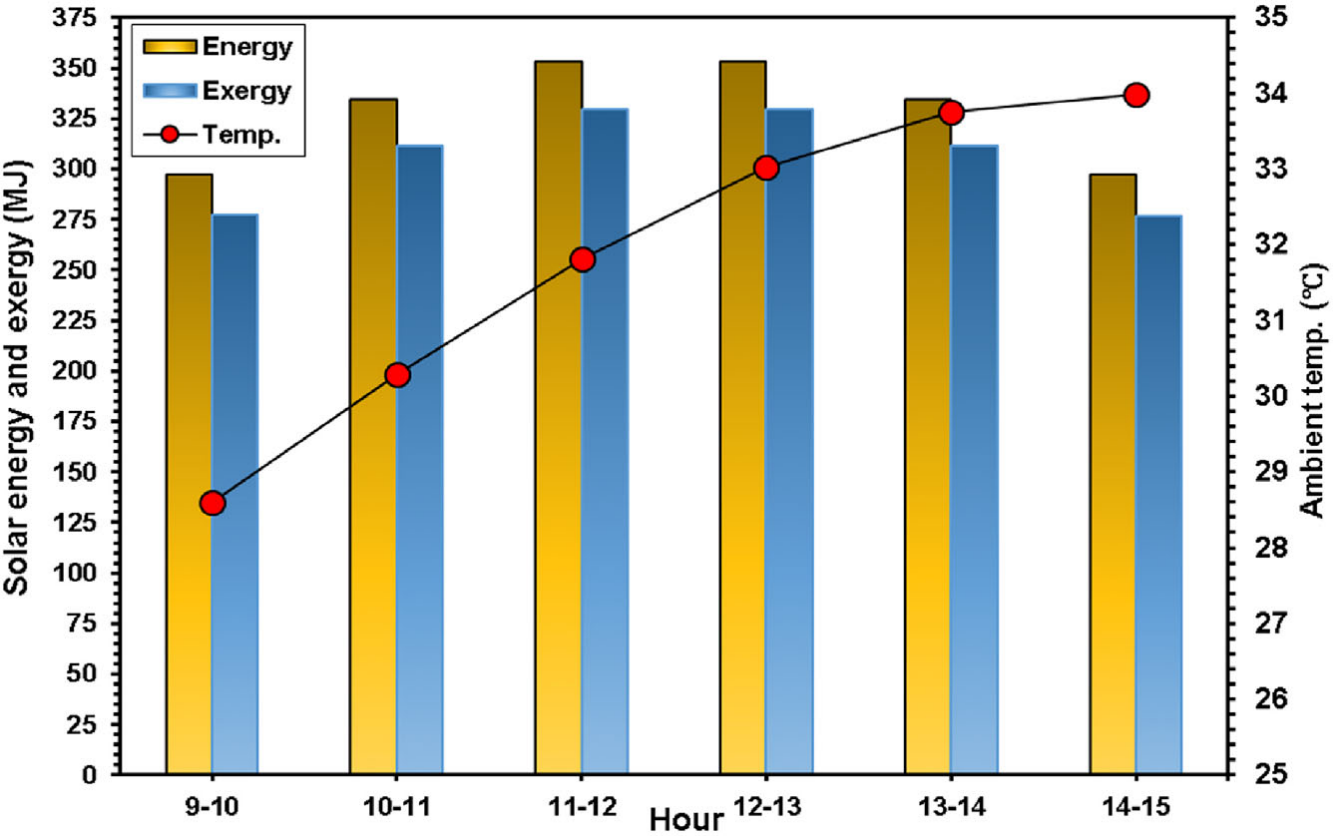
Average hourly solar energy on the FPSCs and ambient temperature.

Figure 3 displays the hourly variation of the solar irradiance on the FPSCs and the ambient temperature (°C) for a day in July. The figure illustrates the time‐dependent changes in solar irradiance intensity and temperature throughout the daytime sunshine period and represents the climatic data used in the system performance analysis. The hourly solar energy and exergy incident on the collector surfaces reached their maximum values between 11:00 and 13:00 at 353.50 and 329.46 MJ, respectively, while the minimum values were observed between 09:00–10:00 and 14:00–15:00 at 297.10 and 276.83 MJ, respectively. Over the entire day, the total radiation and exergy incident on the collector surfaces were 1969.80 and 1836.32 MJ, respectively. Between 14:00 and 15:00, the ambient temperature peaked at 33.99°C, while between 09:00 and 10:00, it dropped to a minimum of 28.59°C. It was observed that solar energy incident on the collector surfaces reached its peak during midday hours and decreased during the morning and afternoon periods. The ambient temperature continued to rise from 09:00 to 15:00 as the incoming solar energy increased, since the high intensity of solar radiation directly contributed to the increase in ambient temperature.

**FIGURE 4:**
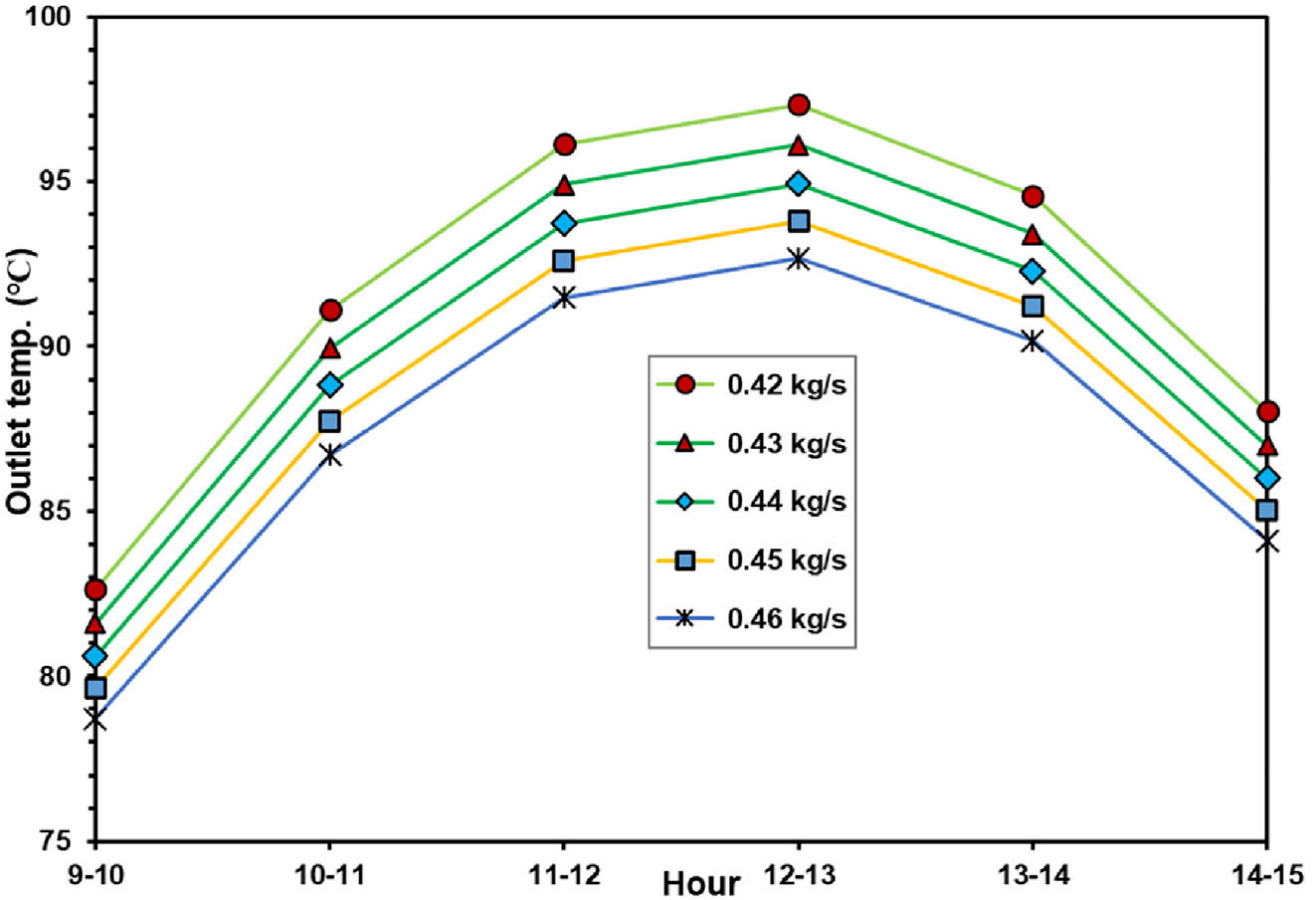
Daily distributions of outlet temperatures of the FPSCs.

Figure 4 illustrates the hourly variation of the outlet temperature (°C) of the FPSC corresponding to different water mass flow rates of 0.42 , 0.43 , 0.44 , 0.45 , and 0.46 kg/s throughout the day. Figure 4 shows the change in collector outlet temperature with increasing flow rate and is used to evaluate the sensitivity of the system performance to flow rate changes. At mass flow rates of 0.42 , 0.43 , 0.44 , 0.45 , and 0.46 , the best outlet temperatures of the collectors were recorded as 97.34°C, 96.11°C, 94.92°C, 93.78°C, and 92.67°C, respectively, between 12:00 and 13:00. Correspondingly, the minimum outlet temperatures were 82.64°C, 81.60°C, 80.61°C, 79.64°C, and 78.72°C between 09:00 and 10:00. For all mass flow rates, the FPSC's outlet temperature reached its maximum during midday, when solar irradiance was highest, and its minimum in the morning due to minimum solar radiation and ambient temperature. Moreover, it is obvious that the outlet temperature of FPSCs decreases as the flow rate increases, while lower mass flow rates result in higher outlet temperatures. This phenomenon is explained by the working fluid's shorter residence time within the FPSCs at greater mass flow rates, which subsequently reduces the amount of time available for efficient heat transfer. This inverse relationship between flow rate and outlet temperature is critical because lower flow rates enhance thermal energy quality for the ORC, whereas higher flow rates reduce temperature but increase the total available thermal mass for energy conversion. From a thermodynamic perspective, the reduction in outlet temperature with increasing mass flow rate occurs because the working fluid spends less time in contact with the absorber plate, reducing the effectiveness of heat transfer. The finite heat transfer coefficient and the fixed collector area limit the amount of energy that can be transferred per unit mass of fluid.

The selected mass flow rate range (0.42–0.46 kg/s) was determined based on the FPSC outlet temperature limitations for safe and efficient ORC operation with R134a. As shown in Figure 4, the outlet temperatures range from approximately 78°C to 97°C across the five flow rates during the day. Flow rates below 0.42 kg/s would cause outlet temperatures to exceed 100°C (the critical temperature limit for R134a under the evaluated pressure conditions), posing safety risks and potential working fluid degradation. Flow rates above 0.46 kg/s result in outlet temperatures below 75°C, where ORC power generation becomes negligible due to insufficient thermal energy quality. Therefore, the 0.42–0.46 kg/s range represents the feasible operating window for the proposed FPSC–ORC system, and the study aims to investigate performance variations within this critical temperature window.

**FIGURE 5:**
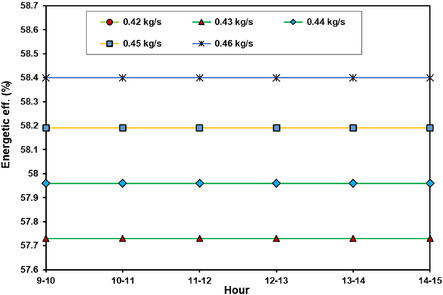
Daily energetic efficiency of the FPSC.

Figure 5 shows how the energy efficiency of FPSCs changes throughout the day at various mass flow rates of 0.42, 0.43, 0.44, 0.45, and 0.46 kg/s. The graphic is used to assess how flow rate effects the thermal efficiency, as well as to show how fluctuations in solar irradiation affect the efficiency of FPSCs. According to the obtained results, the average energy efficiency of the collectors was calculated as 57.49% at 0.42 kg/s, 57.73% at 0.43 kg/s, 57.96% at 0.44 kg/s, 58.19% at 0.45 kg/s, and 58.40% at 0.46 kg/s. According to these findings, the system's energy efficiency steadily improves as the fluid mass flow rate rises because the heat that accumulates on the FPSC's surface is transported more efficiently. Furthermore, energy efficiency did not significantly change during the day (from 9:00 to 15:00) when all mass flow rates were held constant. This implies that short‐term variations in solar radiation throughout the day have no appreciable impact on the collector's energy conversion efficiency and that the system reaches a stable thermal equilibrium for a specific flow rate. As a result, the best mass flow rate choice is emphasized as an important system design element that directly affects the improvement of FPSC energy efficiency. The nearly flat daily trend indicates that FPSC energy efficiency is primarily governed by mass flow rate rather than hourly irradiance fluctuations, which is a beneficial feature for stable system operation. The improvement in FPSC energy efficiency with higher flow rates is explained by the increased heat transfer coefficient at higher Reynolds numbers. As the flow rate increases, the convective heat transfer from the absorber plate to the working fluid becomes more effective, allowing more of the absorbed solar energy to be captured as useful heat rather than being lost to the surroundings.

**FIGURE 6:**
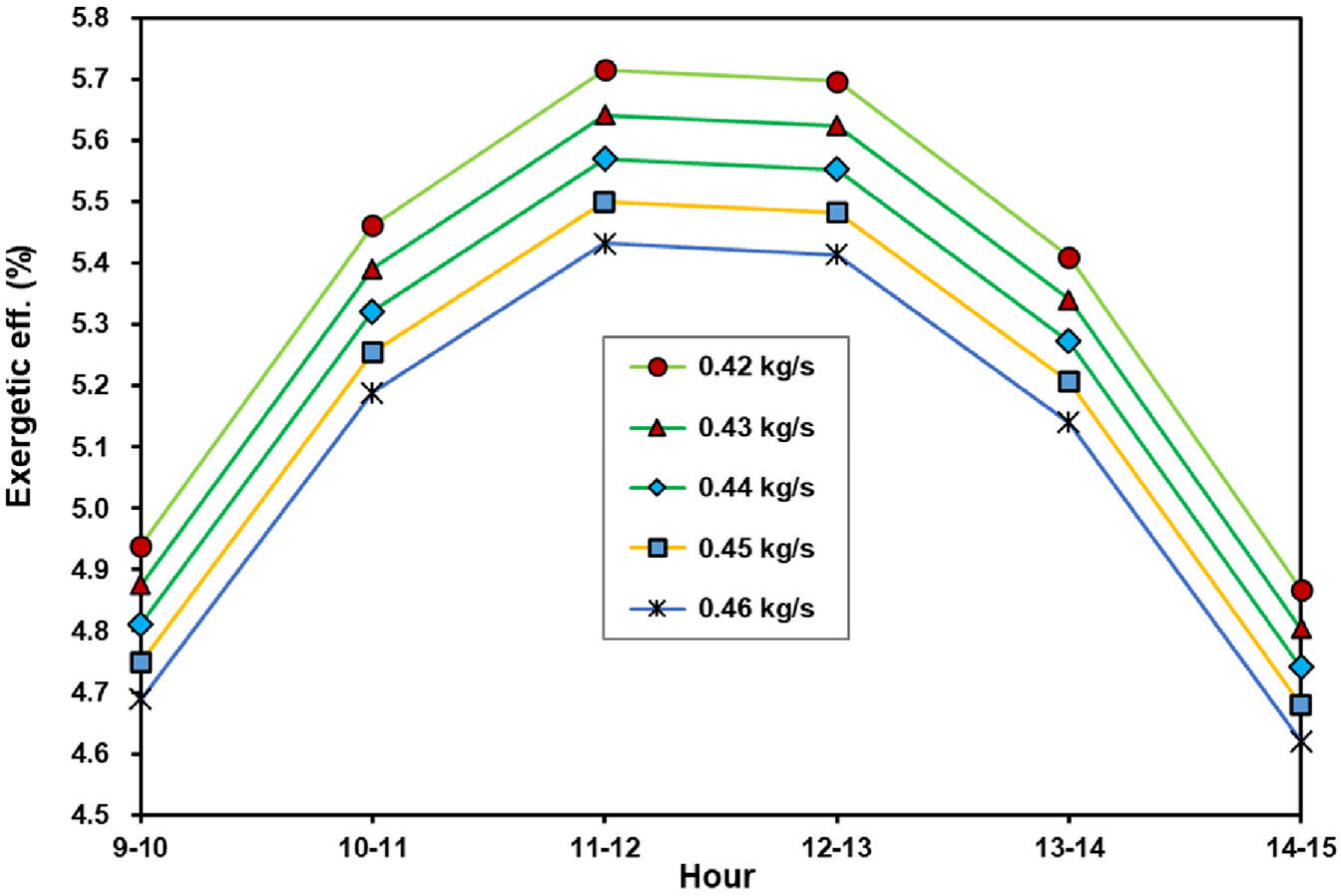
Daily exergetic efficiency of the FPSC.

Figure 6 illustrates how the FPSCs’ exergetic efficiency changed during the day at flow rates between 0.42 and 0.46 kg/s**.** The data show that the FPSC's energetic efficiency rises from morning until midday before starting to decline in the afternoon. For all mass flow rates, the highest exergy efficiency was achieved between 11:00 and 12:00. During this period, the efficiency decreased from 5.72% at 0.42 kg/s to 5.43% at 0.46 kg/s. The lowest exergy efficiencies were recorded between 14:00 and 15:00 as 4.87%, 4.80%, 4.74%, 4.68%, and 4.62% for increasing mass flow rates, respectively. The findings clearly show that energy efficiency decreases with the increase in flow rate. This tendency happens because, at greater flow rates, the fluid spends less time in the collector, and the heat received from solar radiation is transported with a lower temperature difference than the surroundings. The reduction in temperature difference lowers the ratio of usable energy, that is, the exergy efficiency. Unlike the increasing trend observed in energy efficiency, this finding shows that the quality of energy conversion in terms of exergy decreases as the mass flow rate increases. Furthermore, peak exergetic efficiencies for all mass flow rates were seen around midday (11:00–13:00), when solar radiation was at its peak, allowing the collectors to establish thermal equilibrium and produce the most usable energy. Finally, the exergetic efficiency of the FPSC is directly proportional to the intensity of solar radiation while inversely proportional to the flow rate. This shows that while the total amount of energy increases, the quality of that energy declines with increasing flow rates, stressing the essential importance of choosing the ideal flow rate for maximum FPSC performance. The midday peak in exergy efficiency coincides with maximum solar irradiance, confirming that exergy performance is more sensitive to radiation intensity than energy efficiency, which remains relatively flat throughout the day. The decrease in exergy efficiency with higher flow rates is explained by the second law of thermodynamics: Although more energy is collected, the temperature of the collected heat is lower, reducing its quality or useful work potential relative to the ambient environment. Lower temperature differences between the fluid and the surroundings result in smaller Carnot factors, hence lower exergy efficiency.

**FIGURE 7:**
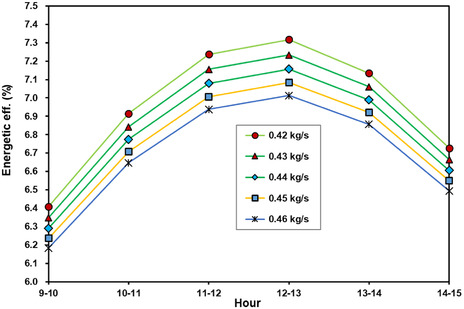
Daily energetic efficiency of the ORC.

Figure 7 represents the energy performance of the ORC powered by thermal energy from FPSCs at various mass flow rates of 0.42, 0.43, 0.44, 0.45, and 0.46 kg/s. The image helps determine the optimal operating settings for the system and shows how various mass flow rates affect the ORC's thermodynamic performance. The ORC system's energy efficiency peaked between 12:00 and 13:00, with efficiencies of 7.32%, 7.23%, 7.16%, 7.08%, and 7.01% for the different mass flow rates, according to the results. The minimum energy efficiencies were obtained between 09:00 and 10:00 as 6.41%, 6.35%, 6.29%, 6.24%, and 6.18%. The ORC's energy efficiency declined as the mass flow rate rose because the water's temperature dropped as it left the collectors. Heat transmission is reduced at higher flow rates because the working fluid has a shorter residence time inside the collector. The best ORC energy efficiency was achieved at noon, when solar irradiation was at its highest and the collector output temperature rose. The ORC efficiency dropped over the remainder of the day as the collector outlet temperature dropped along with the solar irradiation. Overall, the temperature of the incoming water directly affects the energy efficiency of the ORC, but it is reduced when the flow rate is increased. Determining the optimal flow rate is therefore essential to attaining optimal system performance. The ORC energy efficiency follows the same ranking as FPSC outlet temperature, confirming that lower flow rates (0.42 kg/s) provide better thermal‐to‐electric conversion despite producing less total electricity on an hourly basis. The decrease in ORC energy efficiency with increasing flow rate occurs because the lower FPSC outlet temperature reduces the temperature difference between the heat source and the working fluid. According to Carnot efficiency principles, a smaller temperature differential across the evaporator limits the theoretical maximum thermal efficiency, directly reducing the actual energy conversion performance of the ORC.

**FIGURE 8:**
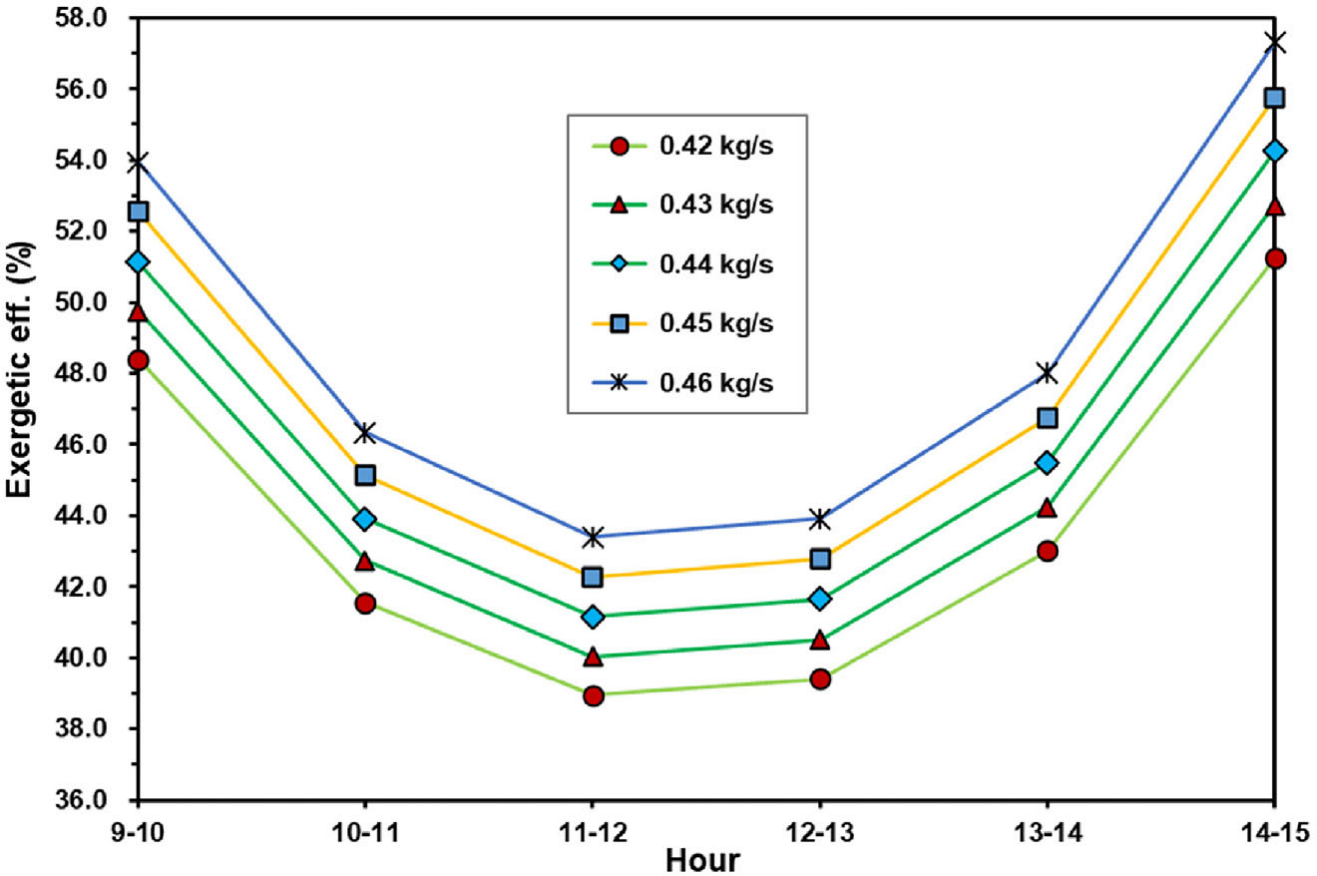
Daily exergetic efficiency of the ORC.

The exergetic efficiency of an ORC powered by thermal energy from FPSCs at different flow rates between 0.42 and 0.46 kg/s is shown in Figure 8. The findings strongly demonstrate that mass flow rate is critical for assessing the energetic effectiveness of the ORC unit. Increased mass flow rates improve the operational fluid's capacity to capture thermal energy, lower temperature variations within heat exchangers, and lessen irreversibilities. The impact becomes most noticeable at a flow rate of 0.46 kg/s, where the system reaches its highest exergetic performance of 57.31%, particularly in the peak thermal hours of 14:00–15:00. On the other hand, as the flow rate drops to 0.42 kg/s, the decreased absorption of heat enhances thermodynamic losses, resulting in a significant decline in exergetic efficiency to 38.95%, with the greatest decrease seen between 11:00 and 12:00, when the thermal intake is lower. The striking increase in ORC exergy efficiency with increasing flow rate (from 38.95% to 57.31%) reveals that higher flow rates significantly reduce thermodynamic irreversibilities within the system, even though the overall energy efficiency shows a slight decrease. This behavior indicates that improved heat‐transfer characteristics and more effective utilization of the available thermal energy occur at higher flow rates, resulting in enhanced exergy recovery and a more thermodynamically optimized ORC operation.

The results demonstrate that the ORC exergetic efficiency gradually improves with increasing mass flow rate and tends to rise under conditions of reduced solar irradiance. Unlike energy analysis, exergy analysis evaluates not only the quantity of energy but also its quality and usefulness relative to environmental conditions. During midday hours, when solar irradiance and collector temperatures reached their highest values, larger temperature gradients caused greater thermal losses and increased thermodynamic irreversibilities, resulting in lower exergy efficiency. In contrast, under lower irradiance conditions observed during the early morning and late afternoon, the system operated with more balanced temperature distributions, leading to more effective energy utilization. Furthermore, increasing the mass flow rate reduced the residence time of the working fluid inside the collector and evaporator, producing a more uniform temperature profile within the heat exchanger. This reduced the temperature mismatch between the heat source and the working fluid, thereby decreasing entropy generation and irreversible heat‐transfer losses. Consequently, the ORC system achieved higher exergy efficiency and more stable thermodynamic performance at higher mass flow rates, despite operating at relatively lower temperatures.

**FIGURE 9:**
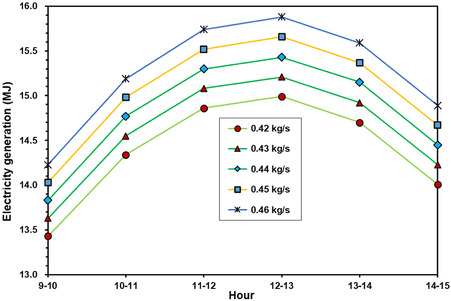
Daily electricity generation of the ORC.

Figure 9 illustrates the hourly electrical power output (MJ) of the ORC system operating with thermal energy provided by FPSCs at mass flow rates of 0.42, 0.43, 0.44, 0.45, and 0.46 kg/s. The figure demonstrates the effect of increasing flow rate on the electricity generation efficiency of the ORC and is utilized to assess the system's daily energy conversion behavior. According to the results, the ORC achieved its maximum hourly electricity production between 12:00 and 13:00, with values of 14.99, 15.21, 15.43, 15.66, and 15.88 MJ for increasing mass flow rates, respectively. Similarly, the minimum production values were recorded between 09:00 and 10:00 as 13.43, 13.63, 13.83, 14.03, and 14.23 MJ. The ORC generated 86.33, 87.62, 88.93, 90.23, and 91.52 MJ of electricity per day for each mass flow rate. During July, the ORC system's overall generation of electricity was 2.676, 2.716, 2.757, 2.797, and 2.837 GJ, according to the chosen mass flow rates. Moreover, in January (lowest solar radiation), the mass flow rate was reduced to 0.175 kg/s and the collector tilt angle was increased to 50° to achieve FPSC outlet temperatures similar to those in July, reaching a maximum of 95.68°C. Under these conditions, the system produced approximately 1.111 GJ of electricity throughout the month. The average electricity generation between July (2.837 GJ at 0.46 kg/s) and January (1.111 GJ at 0.175 kg/s with 50° tilt angle) was calculated as approximately 1.974 GJ. The average monthly energy production of 1.974 GJ corresponds to an annual energy output of approximately 6576 kWh, which was used as the basis for the LCOE calculation.

These findings demonstrate that, while every rise in mass flow rate causes just a slight boost in daily generation of electricity, the cumulative effect over the course of 1 month produces a large total energy gain. Greater mass flow rates allow for increased efficiency in thermal energy consumption, resulting in an increasingly constant and effective monthly power generation from the ORC unit. The results clearly demonstrate that as flow rate increases, so does the ORC's capacity to produce energy. As more heat is transported from the collectors to the ORC during the noon solar irradiation peak, maximum energy generation takes place during that time. However, the system's electricity generation decreased in tandem with the morning and afternoon drops in solar energy. The ORC's total electricity generation, which closely reflects its energy efficiency throughout the day, shows that higher mass flow rates improve power‐generating performance. This highlights the need to figure out the optimal mass flow rate to raise the total energy conversion performance of the system. Although the per‐hour differences in electricity output are relatively modest (approximately 0.2 MJ for every 0.01 kg/s increase in mass flow rate), the cumulative effect over extended operating periods becomes significant. When evaluated on a monthly basis, this gradual increase results in a substantial 0.161 GJ difference between the lowest and highest flow rates, highlighting the strong influence of mass flow rate on the long‐term electrical energy production performance of the ORC system.

**FIGURE 10:**
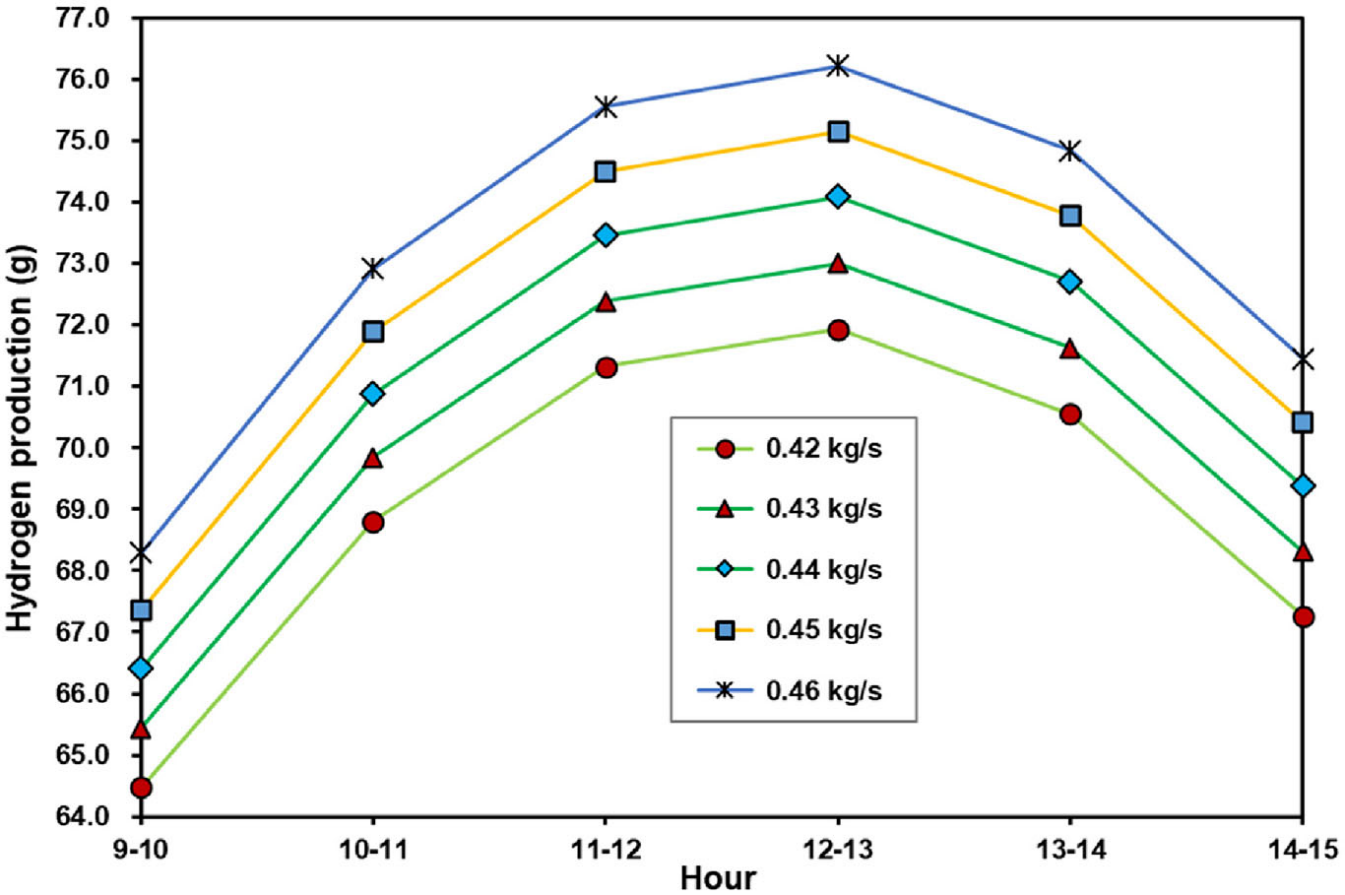
Daily hydrogen production of the PEM.

The thermal‐electrical‐hydrogen coupling in the proposed system operates as follows: Solar irradiance is first converted to thermal energy in the FPSCs. This thermal energy drives the ORC, where a portion is converted to electricity. The electricity is then supplied to the PEM electrolyzer for hydrogen production. Under higher mass flow rates, while the FPSC outlet temperature decreases, the ORC receives greater total thermal mass, resulting in slightly higher electricity generation (Figure 9) and consequently higher hydrogen output (Figure 10). Thus, the coupling is primarily governed by the trade‐off between thermal quality and thermal quantity, with the PEM performance directly following ORC power output under constant electrolyzer efficiency.

Figure 10 illustrates the changes in hydrogen production from the PEM in conjunction with the ORC system, which makes use of the thermal energy absorbed by the FPSCs at flow rates ranging from 0.42 to 0.46 kg/s. Between 09:00 and 10:00, the hydrogen lowest yield was 64.47 g at 0.42 kg/s and 68.30 g at 0.46 kg/s. Solar intensity, mass flow, and hydrogen generation efficiency are directly related. The maximum production levels were observed between 12:00 and 13:00, which corresponded to 71.93, 73.01, 74.08, 75.15, and 76.22 g for gradually increasing flow rates. With the increase in flow rate, the total amount of hydrogen produced each day increased from 414.35 to 439.30 g. In July, the PEM electrolyzer supplied through the ORC‐FPSCs system produced 12.85–13.62 kg of hydrogen, according to the mass flow rate. While the average daily differences between the flow‐rate scenarios seem to be tiny (varying from 414.35 to 439.30 g), they add up notably throughout a 31‐day time. As the mass flow rate rises, an increased and steady heat supplied from the ORC improves the electrolyzer performance, allowing for somewhat higher but constant hydrogen generation. As a result, higher mass flow rates not only increase short‐term efficiency but also help produce a significantly higher monthly hydrogen production, highlighting the significance of improving the thermal‐electrical connection between the ORC and PEM devices. The results demonstrate a steady rise in hydrogen generation as flow rate increases, demonstrating the beneficial effect of higher temperature input on electrolysis performance. This increase might be the consequence of greater heat energy being transferred from the FPSCs to the ORC at higher flow rates, which gives the electrolyzer a stronger electrical power source. During midday, when solar irradiance is at its peak, the system produces the most hydrogen. The FPSCs' output temperature declined in the morning and afternoon as solar radiation decreased, resulting in less hydrogen creation. Overall, hydrogen production followed a pattern similar to the ORC system's power output, demonstrating that larger mass flow rates improve the integrated system's overall energy conversion performance and fuel production capability. Hydrogen production directly mirrors the electricity generation behavior of the ORC system, confirming that PEM electrolyzer performance is strongly and nearly linearly dependent on the ORC power output under constant electrolyzer efficiency assumptions. As the electrical output of the ORC increased with higher solar irradiance and mass flow rates, the hydrogen production rate also increased proportionally throughout the operating period. This close correlation demonstrates the effective thermal–electrical integration between the FPSC, ORC, and PEM subsystems and highlights the importance of stable ORC power generation for continuous hydrogen production.

**FIGURE 11:**
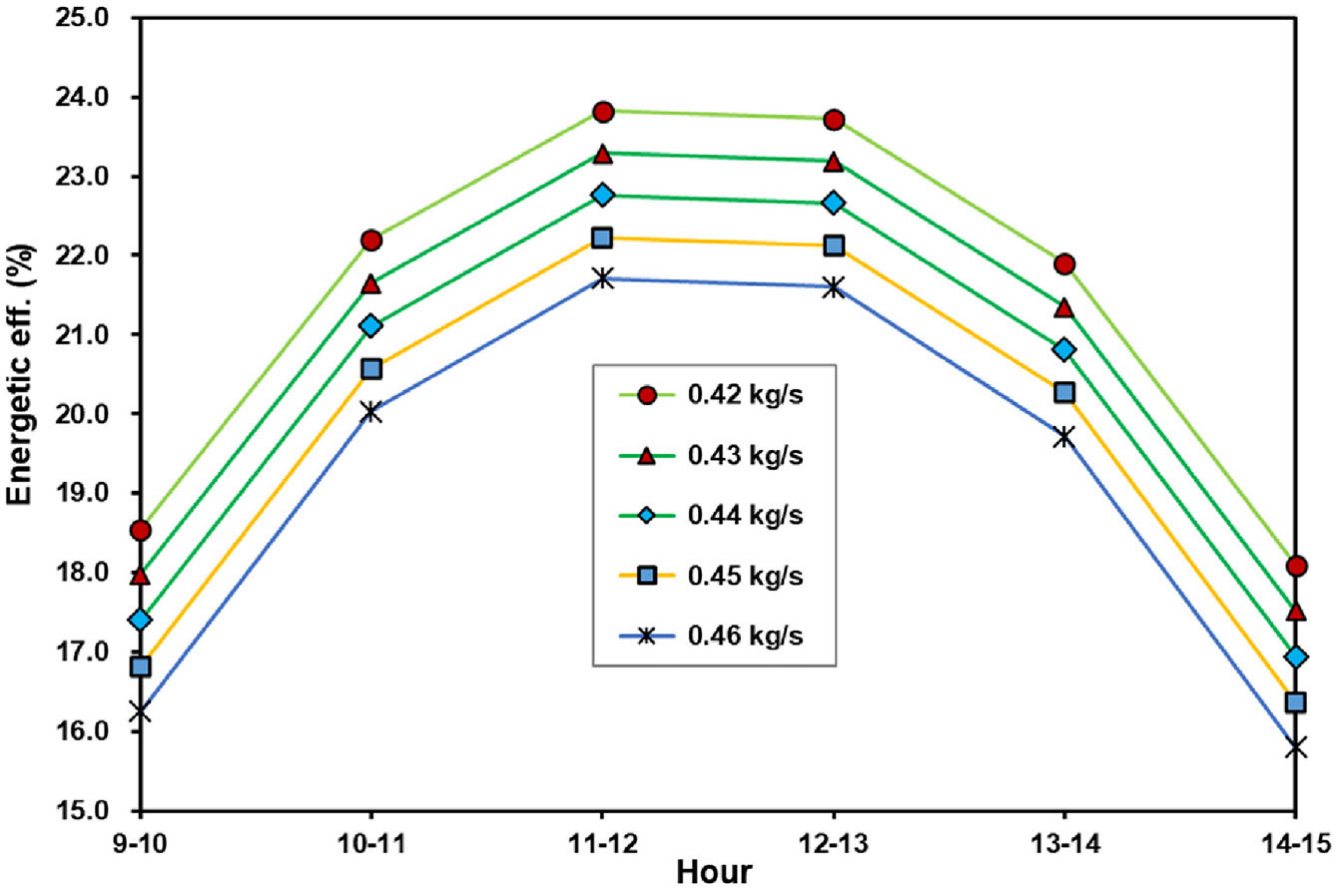
Daily energetic performance of the system.

Figure 11 illustrates the variation in the system's energetic efficiency throughout the day for the ORC system provided by thermal energy from FPSCs at different flow rates ranging from 0.42 to 0.46 kg/s. The results show that the system's energetic efficiency increases from morning to noon and then decreases in the afternoon. The highest energy efficiencies were recorded between 11:00 and 12:00, with values of 23.82%, 23.29%, 22.76%, 22.23%, and 21.71% for 0.42, 0.43, 0.44, 0.45, and 0.46 kg/s, respectively. The lowest efficiencies occurred between 14:00 and 15:00 recorded as 18.10%, 17.52%, 16.94%, 16.37%, and 15.80%. The results show that solar radiation intensity and energy performance are directly proportional. As solar energy peaks around midday, the amount of heat energy transmitted from the collectors to the ORC system reaches its maximum. However, as the mass flow rate increases, the time the working fluid spends for heat transfer in FPSCs decreases, resulting in a small decrease in energy efficiency. As a result, as the mass flow rate increased from 0.42 to 0.46 kg/s, the energy efficiency decreased gradually but only slightly. This finding implies that there is an optimal range of mass flow rates at which the system reaches stable thermal equilibrium. As a result, solar radiation intensity and fluid flow rate are important factors in determining the system's energetic efficiency. The best results are obtained under conditions of low flow rates combined with strong solar irradiance. Overall, the outcomes confirm that the system maintains consistent operation throughout the day, and achieving higher energy conversion efficiency depends greatly on selecting an appropriate mass flow rate. The overall system energy efficiency decreases with increasing mass flow rate because the auxiliary outputs, namely, fruit drying and hot water storage, do not increase at the same rate as the marginal improvement in electricity generation. Although higher flow rates enhance the thermal energy transport and slightly improve ORC power output, this improvement is not sufficiently reflected in the usable thermal outputs of the system. As a result, a larger portion of the additional input energy is not effectively converted into useful end‐use applications, leading to a relative reduction in overall energy efficiency. This indicates that while higher mass flow rates improve certain thermodynamic aspects of the ORC, the multioutput structure of the system limits proportional gains in total energy utilization.

**FIGURE 12:**
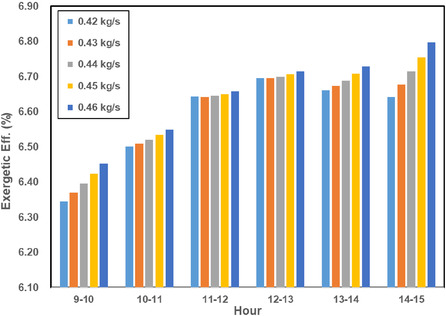
Daily exergetic performance of the system.

Figure 12 shows the hourly distributions of the exergy efficiencies of the system for the ORC utilizing thermal energy from FPSCs at different mass flow rates, changing from 0.42 to 0.46 kg/s. The data showed that the system's energy efficiency was at its lowest in the morning and late afternoon and at its peak around noon. Between 12:00 and 13:00, the maximum exergetic efficiencies were recorded: 6.70%, 6.70%, 6.70%, 6.71%, and 6.72% for mass flow rates of 0.42, 0.43, 0.44, 0.45, and 0.46 kg/s. In contrast. The lowest exergetic efficiencies were seen between 09:00 and 10:00, assessed as 6.35%, 6.37%, 6.40%, 6.42%, and 6.45% respectively. The hourly distribution of the exergetic efficiency reveals a progressive, albeit minor, enhancement caused by the combination of the impacts of boosting solar thermal input and mass flow rate. As the day continues into the late afternoon, the collectors provide higher quality heat, permitting the fluid in use to take in energy more efficiently while marginally decreasing irreversibilities within the process. While the rise is small from 1 h to the next, larger flow rates assist in regulating heat transfer and promoting better thermodynamic circumstances. As a result, the exergetic efficiency climbs moderately during the day, achieving its best values in the late afternoon when both heat capacity and flow‐rate impacts slightly improve exergy use. The nearly constant exergy efficiency (6.70%–6.72%) across all flow rates indicates that the system operates at a stable thermodynamic quality regardless of flow rate, which is a beneficial feature for practical applications. The nearly constant overall exergy efficiency across all flow rates results from a balancing effect: As flow rate increases, irreversibility in the FPSC increases (due to lower outlet temperatures), but irreversibility in the ORC decreases (due to more uniform heat transfer). These two opposing trends compensate each other, leading to stable overall exergy performance.

On the basis of the uncertainty propagation analysis, the overall uncertainties of the system's energetic and exergetic efficiencies were estimated to be approximately ±3.51% and ±3.53%, respectively.

The use of renewable and environmentally friendly energy sources in energy generation is very important to reduce carbon emissions and combat global warming and climate change. Compared to coal‐based systems, renewable energy lowers CO_2_ emissions by 0.034 kg/MJ of thermal energy and 0.404 kg/kWh of electricity [40]. The ORC powered by FPSCs in this study produces thermal energy and electricity without directly releasing CO_2_ into the atmosphere. The use of renewable energy prevents approximately 42.56, 41.78, 40.99, 40.19, and 39.39 kg of CO_2_ emissions per day at mass flow rates of 0.42, 0.43, 0.44, 0.45, and 0.46 kg/s, respectively. Over the month of July, the CO_2_ emission reductions corresponding to each mass flow rate reach 1984.31, 1295.18, 1270.69, 1245.89, and 1221.09 kg, respectively. According to the results, carbon reductions somewhat decrease as a result of variations in heat and power‐generating efficiencies, even though overall energy output increases with greater mass flow rates. This is largely because higher flow rates cause the working fluid to spend less time in the collector, which reduces the temperature differential and, thus, the amount of carbon offset per unit of energy generated. As a result, the proposed integrated system consisting of FPSC‐ORC‐PEM significantly reduces carbon emissions compared to conventional fossil fuel systems. Thus, it provides cleaner and more sustainable energy production and promotes environmental protection. It should be noted that the CO_2_ emission reductions presented above are based on fixed emission factors (0.034 kg/MJ for thermal energy and 0.404 kg/kWh for electricity), which are commonly reported values in the literature. The ±20% variation was adopted as a representative uncertainty range, consistent with commonly accepted practices in energy and environmental assessments. This range reflects the inherent variability in emission factors arising from differences in regional electricity generation mixes, fuel compositions, and methodological discrepancies across data sources. To assess the sensitivity of these results, a brief uncertainty analysis was performed by varying the emission factors by ±20%. Under these variations, the daily CO_2_ reduction for the lowest flow rate (0.42 kg/s) ranges from approximately 34.05 kg to 51.07 kg, while for the highest flow rate (0.46 kg/s) it ranges from 31.51 kg to 47.27 kg. These results indicate that although the absolute values are sensitive to the selected emission factors, the overall trend and environmental benefit remain consistent. It should also be noted that regional variations in electricity carbon intensity may further influence the results. A full life‐cycle assessment (LCA), including manufacturing, operation, and disposal phases, is beyond the scope of the present thermodynamic analysis and is recommended for future work to further enhance the robustness of the environmental evaluation.

The economic assessment is critical for determining the commercial feasibility of the combined ORC–FPSCs–PEM framework because it links incorporated thermal‐electrical‐hydrogen efficiency to real financial results and aids in identifying operational settings that improve the overall profit while assisting long‐term renewable energy generation. The power price for sale is set at 0.118 USD per kWh [41], which serves as the economic foundation for computing the daily and monthly revenue figures utilized in this study. Natural gas of 1 m^3^ generates 10.64 kWh of thermal energy and costs 0.32 USD [42], making it a valuable reference for assessing the economic viability of renewable energy sources. The system produces electricity as well as thermal energy. To analyze its economic benefit, a comparative cost study was undertaken by predicting the daily and monthly costs that would occur if the same quantity of thermal energy were provided by natural gas and the comparable electricity was obtained from the traditional electrical grid. This method offers a clear assessment of the system's total cost‐effectiveness when compared to traditional energy sources. The overall system's economic return decreases slightly as the mass flow rate grows, with 10.90 USD per day and 337.91 USD per month at 0.42 kg/s, 10.72 USD per day and 332.18 USD per month at 0.43 kg/s, 10.53 USD per day and 326.35 USD per month at 0.44 kg/s, 10.34 USD per day and 320.45 USD per month at 0.45 kg/s, and 10.15 USD per day and 314.57 USD per month at 0.46 kg/s, demonstrating that higher flow rates result in slightly lower overall system revenue because lower mass flow rates enable the system to operate at higher thermal energy levels, thereby yielding a marginally greater economic benefit. Based on the electricity generation in July (2.837 GJ, 337.91 USD) and January (1.111 GJ, 132.32 USD), the average monthly revenue was calculated as approximately 235.12 USD, corresponding to an estimated annual revenue of 2,821.44 USD. Considering a 5% nominal interest rate and 3% inflation rate (real discount rate of 1.94%), and a total investment cost of 26,928 USD, the payback period of the system was calculated as 9.73 years. With a system lifetime of 25 years, the project remains economically profitable for approximately 15.3 years after the payback period.

## 5 Model Validation

Validating the created computational framework is critical to delivering accurate and dependable system efficiency evaluations. Model validation, which shows how well mathematical computations and hypotheses match the system's real operational behavior, improves model accuracy, assures the scientific reliability of outcomes, and serves as a firm platform for future optimization and design efforts. Validation is achieved by comparing predictions with information obtained from published research and experiments to verify their accuracy and applicability.

According to a recent study, it was determined that the energetic performance of an FPSC changed between 57.5% and 53% when the solar radiation increased from 100 to 1000 W/m^2^ [43]. Based on Figure 3, the current article found that the collector's energetic performance varied from 57.49% to 58.40% depending on the mass flow rate, while the solar radiation ranged from 735 to 874 W/m^2^.

Another study reported that the exergy efficiency of an FPSC ranged between 3.6% and 5% [44], whereas in this study, it ranged from 4.62% to 5.72%. Additionally, it was found in a separate study that when the source temperature increased from 80°C to 110°C, the thermal efficiency of an ORC using R134a rose from approximately 8.7% to 11.5% [12]. In another work, an ORC system operating with R134a at low temperatures, 16 tests conducted using thermal oil heated to 75–85°C resulted in an efficiency of 6.18% [20]. In the current study, the flat‐plate collectors produced source temperatures ranging from 78.72°C to 97.34°C throughout the day for different flow rates, and the ORC energetic efficiency varied between 6.18% and 7.32%.

Moreover, another study reported that the exergetic efficiency of an ORC running on R134a varied between 72% and 77% [45], while a separate study found it between 35.70% and 45.40% [46]. In the present study, the exergetic performance changed from 38.95% to 57.31%.

Overall, the energy and exergy efficiencies of the two main components of this article, the FPSC and the ORC, were found to be consistent with the data in the literature. This consistency validates the created accuracy and reliability, as well as the system's energy conversion capability, which is in line with previously reported practical and theoretical results.

## 6 Conclusion

This study evaluated the diurnal energetic and exergetic efficiency, as well as the electricity and hydrogen production capacity, of a combined system consisting of FPSCs, an ORC, a PEM electrolyzer, a fruit drying chamber, and a hot water tank. Within the scope of this article and under the specified assumptions, the following conclusions can be drawn.

1. The system was designed and tested for low‐temperature operation, with source temperatures below 100°C that varied during the day based on solar input from the FPSCs.
2. A portion of the produced thermal energy was converted into electricity by the ORC, while the remainder was stored in the tank for later use or directed to the fruit drying chamber.
3. When operated at mass flow rates of 0.42, 0.43, 0.44, 0.45, and 0.46 kg/s, the system produced daily electricity outputs of 86.33, 87.62, 88.93, 90.23, and 91.52 MJ, respectively.
4. During July, the ORC system generated between 2.676 and 2.837 GJ of electricity, depending on the selected mass flow rate.
5. As the mass flow rate increased, daily hydrogen production rose from 414.35 to 439.30 g.
6. Over the month of July, the PEM electrolyzer supplied by the ORC‐FPSC configuration produced between 12.85 and 13.62 kg of hydrogen.
7. The highest daily ORC energy efficiencies were 7.32%, 7.23%, 7.16%, 7.08%, and 7.01%, while the corresponding exergy efficiencies ranged from 38.95% to 57.31%.
8. The use of renewable energy prevented approximately 42.56–39.39 kg of CO_2_ emissions per day as the mass flow rate increased. Throughout July, the CO_2_ reductions associated with each flow rate ranged between 1984.31 and 1221.09 kg.
9. The system's economic return showed a slight decrease with higher flow rates, providing between 10.90 and 10.15 USD per day, corresponding to 337.91–314.57 USD per month, from 0.42 to 0.46 kg/s.
10. The highest hourly energy efficiencies of the system were measured between 11:00 and 12:00, reaching 23.82%–21.71% depending on the increasing mass flow rate, while the maximum exergetic efficiencies remained nearly constant between 6.70% and 6.72%.
11. The payback period of the system was calculated as 9.73 years.

The combined FPSC–ORC–PEM technology looks to be well‐suited for generating electricity from both thermal energy and hydrogen. This dual‐conversion design decreases carbon emissions while increasing the preservation of the environment. Its capacity to generate thermal energy, electricity, and hydrogen simultaneously from solar power provides an appealing option for environmentally friendly energy conversion. By incorporating solar energy, the system reduces environmental impact, increases total energy efficiency, and assists significantly in global attempts to reduce carbon emissions and dependency on fossil fuels. Further studies aimed at making this arrangement more climate‐resilient, energy‐efficient, and economically viable would hasten the adoption of renewable, low‐carbon energy sources while strengthening the world's energy security.

## Nomenclature

**Latin Symbols**
A: Area, (m^2^)
CS: Cost
E: Energy, (kW or MJ)
El: Electricity, (kWh)
Ex: Exergy, (kJ)
f: The annual inflation rate
h: specific enthalpy, (kJ/kg)
HHV: higher heating value of hydrogen, (142 MJ/kg)
i^ı^
: the nominal interest rate
m: Mass, (kg)
ṁ: mass flow rate, (kg/s)
n: Number of independent variables, lifespan time of the system
P: Pressure, (kPa)
R: calculated output parameter (e.g., net power, thermal efficiency, exergy efficiency)
$\dot{Q}$
: Heat transfer rate or, (kW or MJ)
s: specific entropy, (kJ/kg K)
T: Temperature, (°C or K)
u_R_
: Combined uncertainty of the calculated parameter
u_xi_
: Uncertainty of the input variable, (x_i_)
W: Power, (kW or MJ)
Y: total initial investment cost of ORC + FPSCs
x_i_
: Independent input variables
X: Annual profit of ORC + FPSCs
Z: annual operational cost of ORC + FPSCs

**Greek Symbols**
η
: energy efficiency, (%)
ψ
: exergy efficiency, (%)
β
: Working time in a year

**Subscripts**
0: Ambient (dead state)
comp: Compressor
D: depreciation
dest: Destruction
elec: Electrolyzer
eva: Evaporator
F: Faradaic
FPSC: Flat Plate Solar Collector
G: Generator
heat: heating
H_2_
: Hydrogen
in: Input
loss: Loss
M: Maintenance
MD: maintenance and depreciation
m: Mean
net: Net
overall: Overall system
p: price
pump1: Pump 1 (ORC)
pump2: Pump 2
st: Storage tank
tur: Turbine
U: Useful
V: Voltage

## Conflicts of Interest

The authors declare no conflicts of interest.

## Data Availability

Data sharing is not applicable to this article, as no datasets were generated or analyzed during the current study.
